# *Arundina graminifolia* Ameliorates Cisplatin-Induced Acute Kidney Injury via Pathological Targeted Recruitment of Flavonoid Aglycones: A Study Integrating Serum/Kidney Pharmacochemistry and Network Pharmacology

**DOI:** 10.3390/molecules31111951

**Published:** 2026-06-04

**Authors:** Meijia Chen, Yu Zhu, Jianglong Chen, Rujie Zhou, Guang Li

**Affiliations:** 1 Yunnan Branch, Institute of Medicinal Plant Development, Chinese Academy of Medical Sciences & Peking Union Medical College, Xishuangbanna 666100, China; 18043919006@163.com (M.C.);; 2 Yunnan Key Laboratory of Southern Medicine Utilization, Xishuangbanna 666100, China; 3 Institute of Medicinal Plant Development, Chinese Academy of Medical Sciences & Peking Union Medical College, Beijing 100193, China; s2023009012@student.pumc.edu.cn

**Keywords:** *Arundina graminifolia*, acute kidney injury (AKI), cisplatin, UPLC-Q-TOF-MS/MS, pathological targeted recruitment, pharmacodynamic material basis, network pharmacology

## Abstract

Cisplatin-induced acute kidney injury (AKI) poses a significant clinical challenge lacking specific therapeutic drugs. *Arundina graminifolia*, a traditional Dai medicine, exhibits notable renoprotective effects; however, its in vivo pharmacodynamic material basis and molecular mechanisms remain unclear. This study aimed to explore its mechanisms against AKI from the perspective of authentic kidney-migrating components. A cisplatin-induced mouse AKI model was established to evaluate the renoprotective effects of the *A. graminifolia* extract (BYJ) via biochemical markers and histopathology. UPLC-Q-TOF-MS/MS was employed to comparatively analyze the blood and kidney-migrating components between normal and AKI mice. Network pharmacology and molecular docking were subsequently applied to predict and validate the core signaling pathways based on the specific components detected in the injured kidneys. Results showed that BYJ administration significantly ameliorated renal dysfunction, restored antioxidant capacity, and alleviated tubular necrosis. MS analysis identified 93 chemical components in vitro. In vivo tracking revealed a “pathological targeted recruitment” characteristic: only 6 prototype components entered normal kidneys, whereas 16 prototypes penetrated the AKI kidneys, highly enriched in lipophilic flavonoid aglycones such as kaempferol and apigenin. Network pharmacology predicted that these targeted components could potentially interact with 124 key targets (including AKT1, PIK3CA, and EGFR) to putatively exert anti-apoptotic and anti-inflammatory effects via the PI3K-Akt, TNF, and MAPK pathways. Molecular docking confirmed excellent binding affinities between these aglycones and core target proteins (e.g., kaempferol with PIK3CA at −8.9 kcal/mol). Based on actual in vivo distribution, this study reveals the specific accumulation of polyhydroxy flavonoid aglycones in injured kidneys, providing a reliable scientific basis for defining the pharmacodynamic substances of *A. graminifolia*.

## 1. Introduction

Acute kidney injury (AKI) is a severe clinical emergency characterized by a rapid decline in renal function, leading to the rapid accumulation of metabolic waste products and severe fluid and electrolyte imbalances, with a persistently high mortality rate, which remains between 20% and 50% depending on the clinical setting and severity of the injury, even reaching over 50% in critically ill patients requiring renal replacement therapy [1,2]. Among the numerous pathogenic factors contributing to AKI, drug-induced nephrotoxicity accounts for a significant proportion, with cisplatin-induced kidney injury being particularly typical and challenging [3,4]. Cisplatin, a first-line broad-spectrum chemotherapeutic agent for various solid tumors, is specifically taken up and accumulated by organic cation transporters (OCTs) located on the basolateral membrane of renal tubular epithelial cells during its metabolism in vivo. Such high-concentration accumulation directly damages mitochondrial DNA, disrupts the mitochondrial respiratory chain, and triggers an explosive burst of reactive oxygen species (ROS). Excessive ROS not only causes lipid peroxidation of cell membranes but also activates pro-inflammatory signaling pathways, such as nuclear factor-kappa B (NF-κB) and mitogen-activated protein kinases (MAPK), promoting the release of inflammatory cytokines including tumor necrosis factor-alpha (TNF-α) and interleukin-1 beta (IL-1β) [5]. This cascade of reactions ultimately leads to extensive apoptosis and necrosis of renal tubular epithelial cells, resulting in irreversible renal tissue damage. Although the nephrotoxicity of cisplatin is widely recognized, there are currently no approved targeted therapeutic drugs for its prevention and treatment. Clinical management primarily relies on aggressive hydration and symptomatic supportive care. Therefore, identifying active components from natural products capable of intervening in oxidative stress and apoptosis through multiple targets and pathways is of significant clinical importance for the development of anti-AKI drugs.

*Arundina graminifolia* (*D*.*Don*) *Hochr*. (commonly known as bamboo orchid) is a perennial herb of the Orchidaceae family, with a long history of use in the medical systems of ethnic minorities in China (e.g., the Dai people). In the traditional Dai medical theory of “Ya-Jie” (detoxification), *A*. *graminifolia* is considered a key medicinal herb with heat-clearing, detoxifying, and blood-stasis-resolving properties, traditionally used to treat animal and plant poisoning, heat toxins, and nephritic edema [6,7]. Modern phytochemical studies have shown that *A*. *graminifolia* contains a variety of secondary metabolites, mainly including stilbenoids (e.g., resveratrol, pterostilbene), flavonoids (e.g., Quercetin-3-O-rutinoside, quercetin, kaempferol), and phenanthrene derivatives [8,9]. Previous in vitro cellular and in vivo animal experiments have demonstrated that the extracts and some monomeric components of *A*. *graminifolia* possess significant free radical scavenging, anti-inflammatory, and target organ protective biological activities against oxidative damage [10]. However, current research on *A*. *graminifolia* intervening in renal injury mainly focuses on the overall pharmacodynamic observation of the extract or in vitro evaluation of single components. The actual pharmacodynamic material basis and multi-target regulatory network responsible for its anti-AKI effects in vivo remain to be systematically elucidated.

Elucidating the pharmacodynamic material basis of traditional Chinese medicine (TCM) and ethnomedicine is critical for realizing their modernized application. Traditional material basis research often focuses on the identification and isolation of chemical components in vitro. However, upon oral administration, highly abundant in vitro components (e.g., macromolecular flavonoid glycosides) are often hindered by their large molecular weight and high polarity, leading to restricted gastrointestinal absorption or extensive biotransformation mediated by gut microbiota and hepatic enzymes. Therefore, high-abundance components in vitro may not necessarily enter the bloodstream directly, let alone cross the vascular endothelial barrier to distribute to the target lesion tissue. In recent years, network pharmacology, as an emerging systems biology tool, has been widely applied to predict the multi-target mechanisms of TCM interventions in diseases. Nevertheless, in practical applications, conventional network pharmacology analysis often directly uses all the components identified in vitro as the basis for target prediction, without considering the absorption, distribution, metabolism, and excretion (ADME) processes of the drugs in vivo. Such “static prediction” lacking pharmacokinetic data support is prone to introducing interference from unabsorbed components, leading to predicted results that deviate from the true in vivo mechanism of action, resulting in a high “false positive” rate.

Based on the above background, a more rigorous research strategy involves comprehensively integrating in vivo pharmacokinetic distribution with network pharmacology. High-resolution liquid chromatography-tandem mass spectrometry (e.g., UPLC-Q-TOF-MS/MS), owing to its extremely high sensitivity and resolution, has become an effective tool for profiling trace migrating components of TCM in vivo. Therefore, this study aims to systematically investigate the pharmacodynamic material basis and mechanism of action of *A*. *graminifolia* against cisplatin-induced AKI from the perspective of the actual components distributed into the kidney in vivo.

In this study, a cisplatin-induced mouse AKI model was first established to evaluate the protective effect of *A*. *graminifolia* extract (BYJ) on impaired renal function. Secondly, UPLC-Q-TOF-MS/MS technology was employed to comparatively analyze the differences in blood-absorbed and kidney-distributed components between normal mice and AKI model mice, exploring the distribution patterns of BYJ active components locally in the injured kidney. Finally, strictly based on the migrating components actually detected in the injured kidney of AKI mice, combining network pharmacology analysis and computer-aided molecular docking, the key targets and potential signaling pathways involved in its intervention against cell apoptosis and inflammatory responses were investigated. This study not only provides objective experimental data to elucidate the true pharmacodynamic material basis of *A*. *graminifolia* against AKI but also offers reference and validation for mechanism research of TCM and ethnomedicine based on actual in vivo distribution characteristics.

## 2. Results

### 2.1. In Vitro Chemical Composition Identification of A. graminifolia

**Figure 1:**
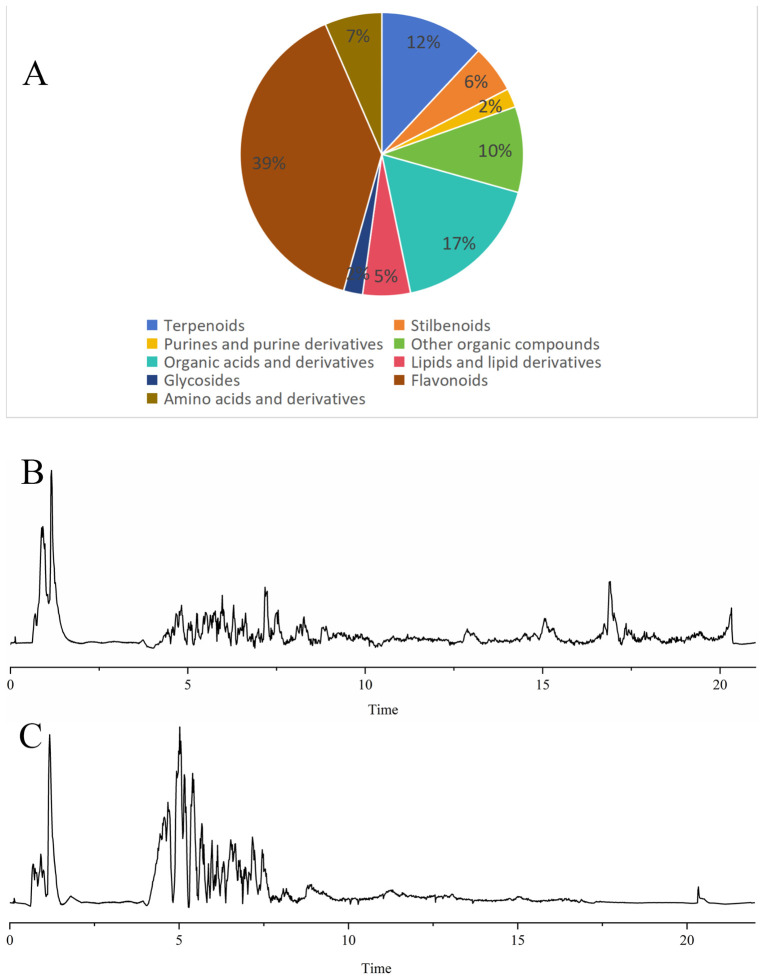
UPLC-Q-TOF-MS/MS identification and classification of chemical components in *Arundina graminifolia* extract (BYJ) in vitro. (**A**) Pie chart illustrating the chemical classification of the 93 identified secondary metabolites (mainly including flavonoids, organic acids, terpenoids, and stilbenes). (**B**) Total ion chromatogram (TIC) of BYJ in positive ion mode. (**C**) TIC of BYJ in negative ion mode.

To comprehensively characterize the chemical composition of the BYJ extract, UPLC-Q-TOF-MS/MS was employed. The total ion chromatograms (TIC) in positive and negative ion modes are presented in Figure 1B and Figure 1C, respectively. During the data processing stage, common endogenous primary metabolites (e.g., amino acids, basic lipids) were excluded to focus on potentially bioactive secondary metabolites, thereby establishing a foundation for tracking in vivo migrating components.

**Figure 2:**
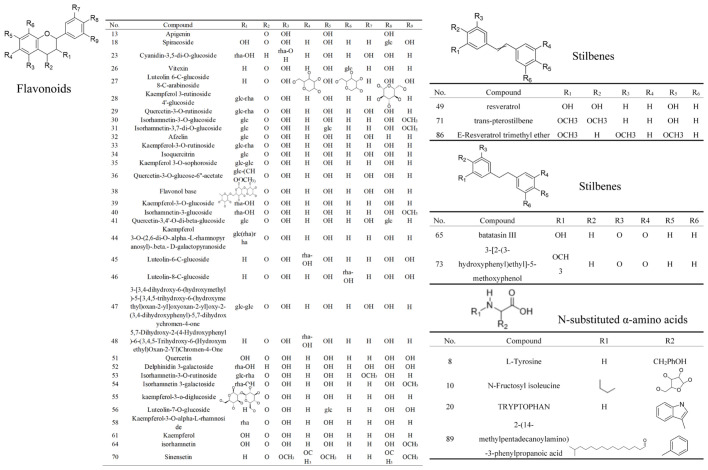
Flavonoid, stilbenoid and N-substituted α-amino acid skeleton compounds.

**Figure 3:**
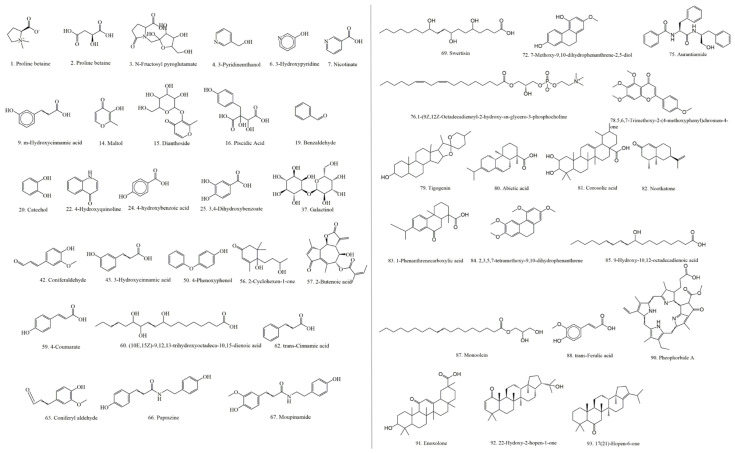
Compounds with other unclassified skeletons.

Based on comparisons with reference standards, exact molecular weights (mass error < 5 ppm), MS/MS fragmentation patterns, and online database matching, a total of 93 chemical components were identified in vitro. These metabolites can be broadly classified into nine major groups: flavonoids (36), organic acids and derivatives (16), terpenoids (11), amino acids and derivatives (6), stilbenoids (5), lipids and lipid derivatives (5), purines and purine derivatives (3), glycosides (2), and other organic compounds (9). The proportional distribution of these chemical classes is shown in Figure 1A. Detailed identification information (retention times, precursor ions, and fragment ions) is provided in the Supplementary Materials (Table S1). The structural skeletons of flavonoids, stilbenes, and N-substituted α-amino acids are illustrated in Figure 2, while other skeleton compounds are presented in Figure 3.

#### 2.1.1. Identification of Flavonoids

A total of 36 flavonoid components were identified in BYJ. The mass spectral cleavage of flavonoid aglycones typically involves Retro Diels-Alder (RDA) cleavage and the loss of neutral molecules such as CO and H_2_O [11]. Under MS conditions, flavonoid glycosides are prone to glycosidic bond cleavage, yielding characteristic fragments resulting from the loss of 162 Da (glucosyl), 146 Da (rhamnosyl), or 308 Da (rutinosyl) [12].

**Figure 4:**
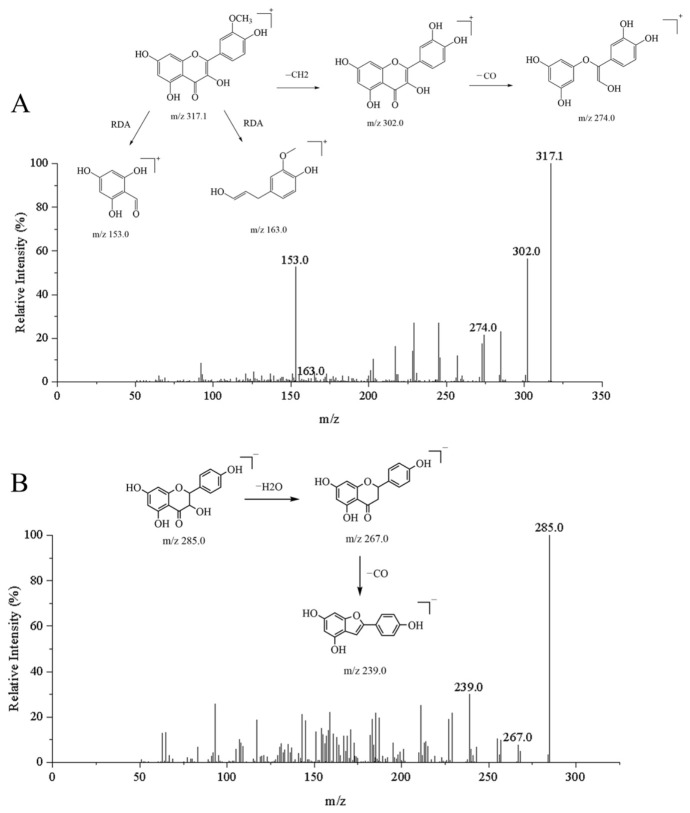
Representative MS/MS fragmentation pathways of core flavonoids. (**A**) MS/MS fragmentation analysis of isorhamnetin. (**B**) MS/MS fragmentation analysis of kaempferol.

Taking isorhamnetin and kaempferol as examples: Peak 70 was identified as isorhamnetin by comparing it with the reference standard. Its MS1 spectrum exhibited a quasi-molecular ion at *m*/*z* 317.1 [M+H]^+^ (C_16_H_13_O_7_^+^). As shown in Figure 4A, the precursor ion lost a CH_3_ radical to produce a fragment at *m*/*z* 302.0 [M+H-CH_3_]^+^, followed by the loss of CO to generate *m*/*z* 274.0 [M+H-CH_3_-CO]^+^. Concurrently, 1,2A and 1,3A RDA cleavages generated characteristic fragments at *m*/*z* 153.0 and *m*/*z* 163.0. Peak 64 was identified as kaempferol (*m*/*z* 285.0 [M+H]^+^, C_15_H_11_O_6_^+^). As shown in Figure 4B, the loss of H_2_O and CO yielded fragments at *m*/*z* 267.0 and 239.0, respectively. Furthermore, in negative ion mode, the precursor ion at *m*/*z* 447.1 generated the same characteristic fragments as peak 64, identifying peak 39 as kaempferol-3-O-glucoside [13]. Similarly, peaks 51 and 26 were identified as quercetin and vitexin, respectively.

#### 2.1.2. Identification of Stilbenes

Stilbenes are critical characteristic components of BYJ, and 5 such compounds were identified in this study. In negative ion mode ([M-H]^−^), their fragmentation behaviors exhibited distinct commonalities. Driven by the stable conjugated system formed by the double bond and benzene rings, the phenoxide anion tends to undergo cleavage at the ortho C-C bond.

**Figure 5:**
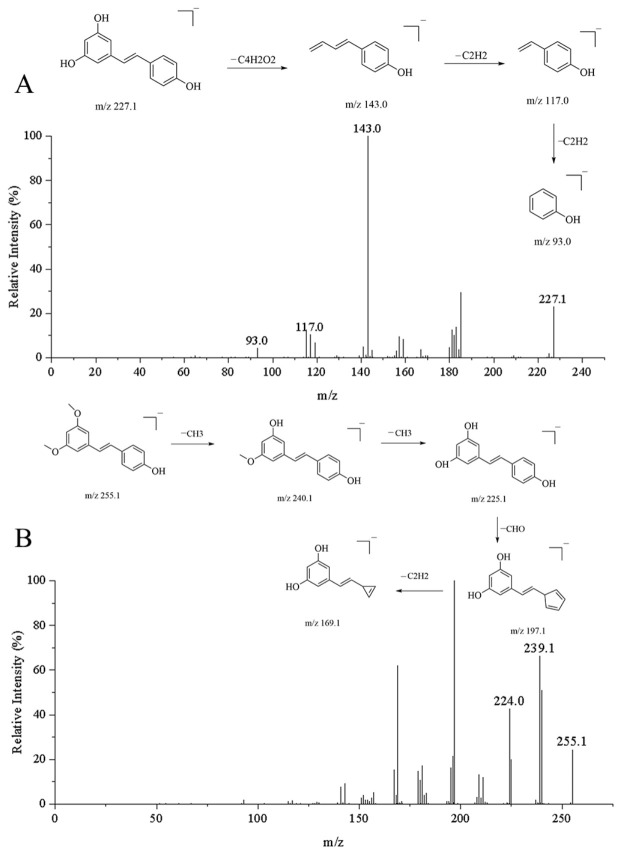
Mass spectrometric analysis of stilbenoids. (**A**) Mass spectrometric identification of resveratrol. (**B**) Mass spectrometric identification of pterostilbene.

Taking resveratrol as an example, peak 49 showed a quasi-molecular ion at *m*/*z* 227.1 [M-H]^−^, corresponding to the formula C_14_H_11_O_3_^−^. Under collision-induced dissociation (CID), characteristic fragments at *m*/*z* 143.0, 117.0, and 93.0 were formed. The *m*/*z* 143.0 fragment resulted from the cleavage of the bond between the benzene ring and the vinyl group (neutral loss of C_4_H_2_O_2_, 84 Da). Subsequently, *m*/*z* 143.0 further lost C_2_H_2_ (26 Da) to form *m*/*z* 117.0, which then lost another C_2_H_2_ to yield a stable negative ion at *m*/*z* 93.0 containing a single benzene ring (Figure 5A) [14]. Additionally, peak 71 was identified as pterostilbene (*m*/*z* 255.1 [M-H]^−^, C_16_H_15_O_3_^−^), presenting a fragmentation pathway involving sequential losses of CH_3_, CHO, and C_2_H_2_ (Figure 5B).

#### 2.1.3. Identification of Nucleosides and Organic Acids

**Figure 6:**
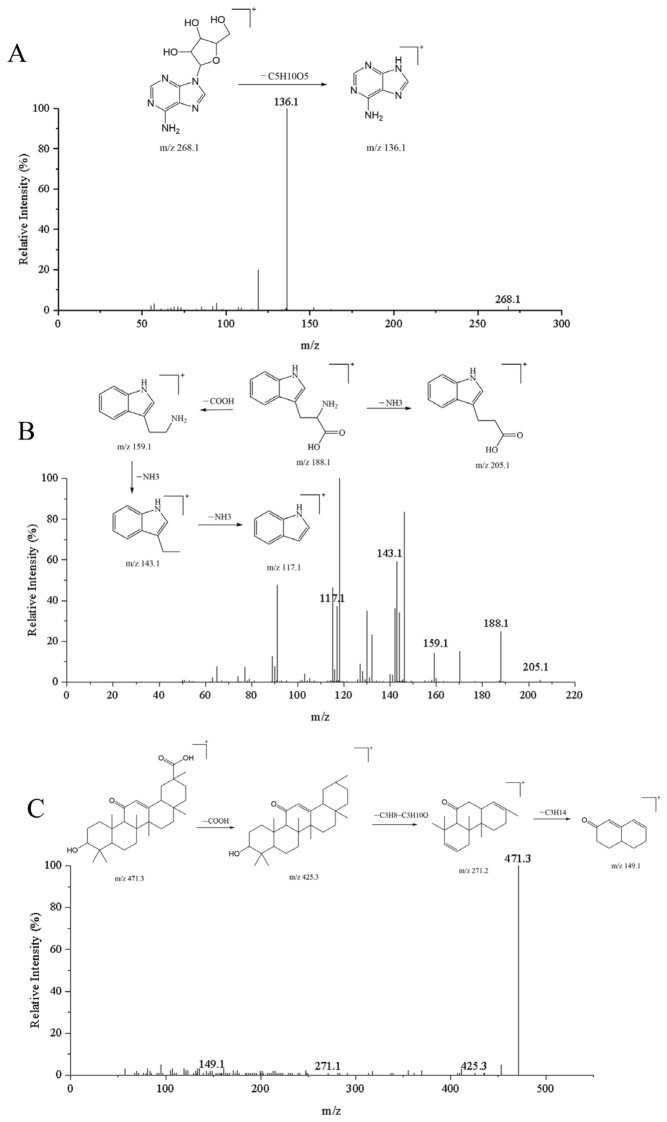
Mass spectrometric analysis of nucleosides, terpenoids and alkaloids. (**A**) Mass spectrometric identification of adenosine. (**B**) Mass spectrometric identification of tryptophan. (**C**) Mass spectrometric identification of glycyrrhetinic acid.

Three nucleosides were identified. Peak 11 displayed a quasi-molecular ion at *m*/*z* 268.1 [M+H]^+^, yielding a fragment at *m*/*z* 136.1 [M+H–C_5_H_8_O_4_]^+^, which is consistent with the reported MS/MS fragments of adenosine (Figure 6A) [15,16].

A total of 25 organic acids were identified, including 11 amino acids. For natural α-amino acids in positive ion mode, typical cleavages included the loss of the free α-amino group (-NH_3_, 17 Da) and decarboxylation (-CO_2_, 44 Da). For instance, peak 20 (*m*/*z* 205.1 [M+H]^+^) was identified as tryptophan based on its fragmentation cascade (loss of NH_3_ to *m*/*z* 188.1, followed by the loss of CO_2_ to *m*/*z* 159.1) (Figure 6B). In negative ion mode, peak 2 (*m*/*z* 133.0 [M-H]^−^) was deduced to be malic acid [17].

#### 2.1.4. Identification of Terpenoids

Terpenoid fragmentation is dominated by the cleavage of the carbon skeleton based on isoprene units. Compound 91 was detected in positive ion mode at 16.893 min with a quasi-molecular ion at *m*/*z* 471.3473 [M+H]^+^ (C_30_H_47_O_4_^+^). It was identified as glycyrrhetinic acid based on consecutive losses of CH_2_O_2_, C_5_H_8_, CO, and CH_2_, yielding characteristic fragments at *m*/*z* 425.3426, 271.2064, and 149.0961 (Figure 6C).

### 2.2. BYJ Significantly Ameliorates Cisplatin-Induced Acute Kidney Injury (AKI)

#### 2.2.1. Improvement of Clinical Signs and Macroscopic Kidney Indices

**Figure 7:**
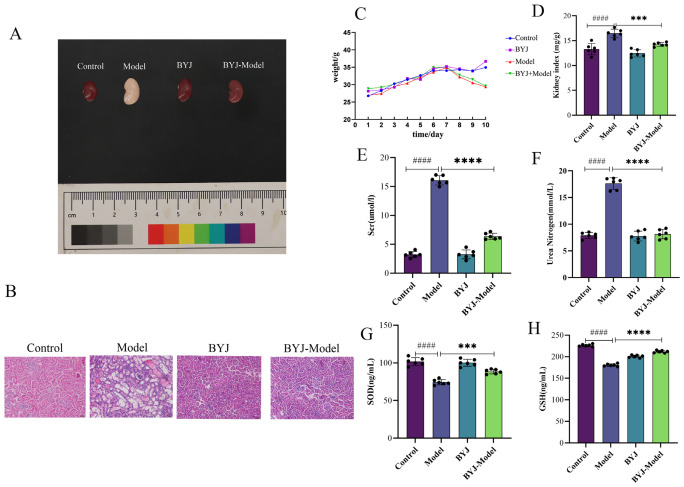
Pharmacodynamic evaluation of BYJ in alleviating cisplatin-induced acute kidney injury (AKI) in mice. (**A**) Macroscopic appearance of bilateral kidneys from each group. (**B**) Representative H&E staining of renal tissues (magnification 200×; arrows indicate necrotic tubules and proteinaceous casts). (**C**) Body weight changes during the experiment. (**D**) Kidney index. (**E**,**F**) Renal function markers: serum creatinine (Scr) and blood urea nitrogen (BUN) levels. (**G**,**H**) Local oxidative stress markers in renal tissues: superoxide dismutase (SOD) and glutathione (GSH) levels. Data are presented as mean ± SD (*n* = 6), and individual biological replicates are overlaid as scatter dots on the bar graphs. Statistical significance was determined using one-way ANOVA followed by Tukey’s post-hoc test. ^####^ *p* < 0.0001 vs. Control group; *** *p* < 0.001, **** *p* < 0.0001 vs. Model group.

During the 10-day administration period, mice in the Control and BYJ-Normal groups showed steady weight gain, glossy coats, and normal activity, indicating excellent in vivo safety of BYJ at the given dose. After intraperitoneal injection of cisplatin on day 7, mice in the Model group rapidly exhibited acute toxic stress signs (e.g., lethargy, reduced activity) and significant weight loss. Macroscopically (Figure 7A), the bilateral kidneys of the Model group were noticeably enlarged, pale, and locally congested. This pale discoloration is a characteristic macroscopic manifestation of cisplatin-induced AKI, primarily resulting from renal microcirculatory dysfunction (ischemia), massive tubular necrosis, and severe interstitial edema. These typical morphological changes are highly consistent with findings from previous studies using the same animal model [18,19]. The kidney index (kidney weight/body weight ratio) was significantly elevated compared to the Control group (*p* < 0.001), indicating severe renal edema and acute inflammation. Following continuous BYJ intervention (BYJ-Model), clinical toxicity signs were effectively alleviated. The swelling and congestion of the kidneys were visibly reduced, and the kidney index decreased significantly compared to the Model group (*p* < 0.01).

#### 2.2.2. Amelioration of Renal Microscopic Histopathology

Hematoxylin and Eosin (H&E) staining provided microscopic cytological evidence. As shown in Figure 7B, the Control and BYJ-Normal groups exhibited an intact renal architecture with tightly arranged tubular epithelial cells and clear brush borders. Conversely, the Model group presented typical acute tubular necrosis (ATN) characteristics: extensive tubular dilation, severe vacuolar degeneration of the epithelial cells, loss of brush borders, massive necrosis, and the formation of dense pink proteinaceous casts. In the BYJ-Model group, cisplatin-induced structural destruction was effectively restricted. The number of necrotic and detached epithelial cells was significantly reduced, brush border structures were partially preserved, and proteinaceous casts nearly disappeared, demonstrating BYJ’s efficacy in maintaining renal microscopic integrity.

#### 2.2.3. Restoration of Renal Function and Local Oxidative Stress Parameters

Regarding renal function markers (Figure 7D,E), cisplatin toxicity led to a severe decline in the glomerular filtration rate (GFR), evidenced by serum Scr and BUN levels in the Model group being significantly higher than those in the Control group (*p* < 0.001). Consistent with the morphological improvements, BYJ administration effectively reversed this trend, significantly lowering Scr and BUN levels (*p* < 0.01).

Mechanistically, cisplatin induces mitochondrial dysfunction and severe reactive oxygen species (ROS) bursts. Biochemical analysis of renal tissue homogenates (Figure 7F–H) revealed that core antioxidant defense enzymes, such as superoxide dismutase (SOD), and endogenous antioxidants (GSH) were severely depleted in the Model group (*p* < 0.001). Following BYJ intervention, local renal antioxidant capacity was reactivated, and the SOD and GSH levels were significantly recovered (*p* < 0.01).

### 2.3. Screening and Identification of Blood and Kidney-Migrating Components Based on Multi-Dimensional Matrix Comparison

To overcome the common bottleneck of low compound concentrations and strong endogenous matrix interference in in vivo studies, a multi-dimensional cross-comparison method (“Control vs. BYJ-Normal” and “Model vs. BYJ-Model”) was constructed. This approach aimed to accurately identify the migrating components of BYJ in the blood and kidneys, exploring their delivery rules and “pathological targeted recruitment” characteristics.

#### 2.3.1. Distribution Characteristics in the Normal Physiological State

**Figure 8:**
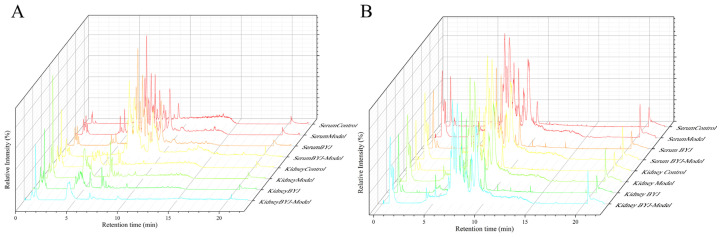
Total ion chromatograms of four animal groups in serum and kidney. (**A**) Total ion chromatogram in positive ion mode. (**B**) Total ion chromatogram in negative ion mode.

In healthy mice (BYJ-Normal group), after strictly subtracting endogenous background signals from blank serum, 17 prototype components and 9 Phase I/II metabolites were identified in the serum, covering flavonoids, phenolic acids, and triterpenoids. This indicates that the BYJ extract has a good basis for gastrointestinal absorption. The total ion chromatograms (TIC) overlay for each group is shown in Figure 8.

However, in the kidneys of the BYJ-Normal group, only 6 prototype components and 8 metabolites were identified. This suggests that under normal physiological conditions, the intact renal microvascular endothelium strictly filters molecules, preventing the majority of macromolecular and highly lipophilic compounds (as well as unconjugated free aglycones) from penetrating normal renal tissues in large quantities.

#### 2.3.2. Component Analysis of “Pathological Targeted Recruitment” in the AKI State

Cisplatin-induced AKI triggers systemic inflammation and abnormally increases renal microvascular permeability. By comparing the renal metabolic profiles of normal and diseased mice, a remarkable chemotactic distribution phenomenon was discovered: while only limited molecules crossed the barrier in normal kidneys, the number of accumulated prototypes increased to 16 in cisplatin-damaged kidneys, accompanied by 8 specific metabolites.

Notably, the newly accumulated molecules in the injured kidneys were highly concentrated in highly lipophilic flavonoid aglycones (such as kaempferol, apigenin, and isorhamnetin) and certain lipid molecules. This phenomenon highlights a “pathological targeted recruitment.” The mechanism likely involves cisplatin disrupting the endothelial barrier and causing extensive lipid peroxidation of tubular cell membranes, altering the local microenvironment. This pathological microenvironment may confer a strong “pathological affinity” to lipophilic flavonoid aglycones, driving their directional accumulation. The dynamic evolution and distribution mapping of these components are comprehensively summarized in Table 1.

### 2.4. Structural Dynamic Evolution of Migrating Components in Vivo

**Figure 9:**
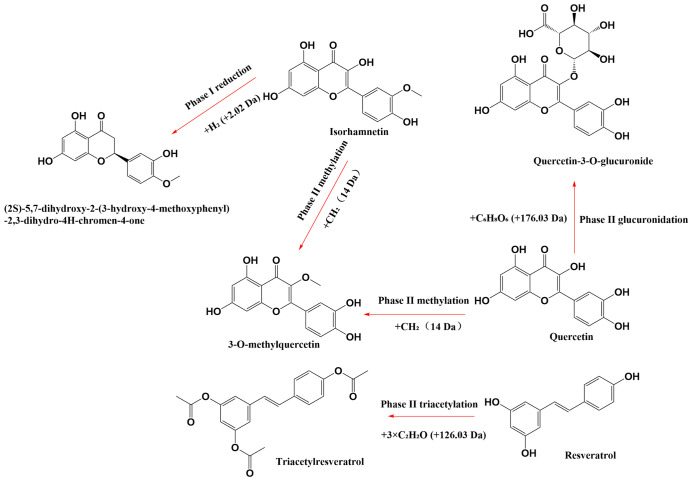
In vivo transformation mechanism of isorhamnetin and quercetin.

**Figure 10:**
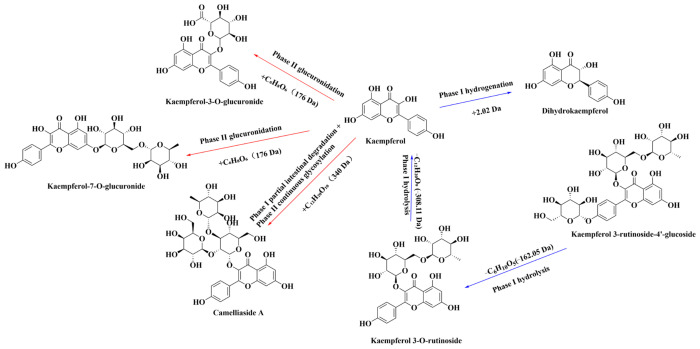
In vivo transformation mechanism of kaempferol. Red arrows indicate Phase II metabolic pathways or a combination of Phase I and Phase II pathways. Blue arrows indicate Phase I metabolic pathways.

**Figure 11:**
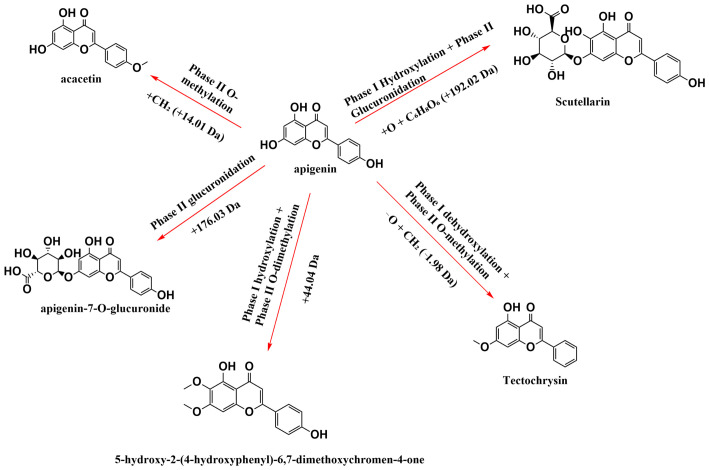
In vivo transformation mechanism of apigenin. Red arrows indicate Phase II metabolic pathways or a combination of Phase I and Phase II pathways.

The MS correlation analysis indicated that the core flavonoids in BYJ (quercetin, kaempferol, isorhamnetin, and apigenin) exhibited significant metabolic transformation in vivo. Macromolecular flavonoid O-glycosides (e.g., Quercetin-3-O-rutinoside), which are abundant in vitro, are rarely absorbed directly as prototypes. Instead, they undergo widespread Phase I hydrolysis and reduction mediated by gut microbiota (e.g., β-glucosidases), shedding their sugar moieties to form highly lipophilic free aglycones (Figure 9, Figure 10 and Figure 11).

Upon entering the systemic circulation and distributing to the liver and targeted organs (kidneys), these free aglycones undergo extensive Phase II conjugation (glucuronidation and methylation), driven by host enzymes such as UGTs (UDP-glucuronosyltransferases) and COMT (catechol-O-methyltransferase). For example, apigenin exhibits high metabolic diversity, potentially converting to acacetin via methylation or further transforming into baicalin.

Furthermore, compared to the normal state, the AKI pathological model altered specific metabolic pathways. Characteristic stilbenes, such as resveratrol, were specifically detected as triacetylated metabolites (triacetylresveratrol) in the AKI state, reflecting possible fluctuations in acetylase activity under acute stress. This biotransformation pathway—from “macromolecular glycosides” hydrolyzed in the gut to “free aglycones,” and subsequently converted to “conjugated metabolites” in the target organ—elucidates the in vivo delivery rules of the active components of BYJ.

### 2.5. Network Pharmacology Mechanism Prediction and Molecular Docking Verification Based on Authentic Kidney-Migrating Components

#### 2.5.1. Therapeutic Core Component Screening and Target Network Construction

The core of targeted therapy lies in potentially modulating the pathological microenvironment rather than putatively exerting non-specific effects on normal tissues. Although a few components were detected at basal levels in normal kidneys, the 24 recruited components (16 prototypes and 8 metabolites) exhibited significant and specific upregulation exclusively in the AKI pathological state, making them the authentic material basis.

To construct the network pharmacology model, we strictly defined the input boundaries based on this actual in vivo targeted exposure. Recent pharmacological studies emphasize that phase II metabolites and structural derivatives enriched in target organs frequently act as the direct pharmacological effectors in vivo. Therefore, we comprehensively evaluated these 24 components and applied a stringent filtering strategy. Specifically, basal endogenous metabolites (e.g., amino acids), components with extremely low relative abundance, and those lacking identifiable protein targets in existing pharmacological databases were excluded to minimize prediction noise. Following this rigorous selection, exactly 18 therapeutic core components—encompassing structurally intact prototypes (e.g., kaempferol, Quercetin-3-O-rutinoside, apigenin, resveratrol) and their key bioactive metabolites (e.g., apigenin 7-O-glucuronide, triacetylresveratrol)—were established as the input foundation (The detailed list is provided in Supplementary Table S2).

To further validate the “pathological targeted recruitment” of these 18 finalized core components beyond mere qualitative presence (+/−), we conducted a semi-quantitative analysis based on their relative MS peak areas. As illustrated in the heatmap (Supplementary Figure S2), these components exhibited dramatically enhanced relative abundances in the AKI-damaged kidneys compared to the normal kidneys. This compelling semi-quantitative evidence robustly confirms that the pathophysiological microenvironment actively drives the massive, targeted accumulation of these specific active metabolites, firmly bridging the in vivo exposure with the subsequent network pharmacology predictions.

**Figure 12:**
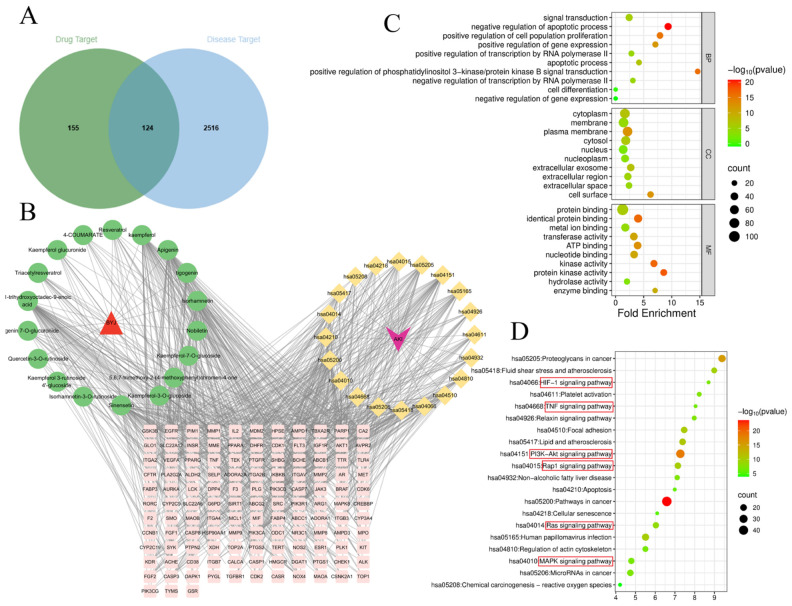
Network pharmacology analysis of the core kidney-recruited components of *Arundina graminifolia* against cisplatin-induced AKI. (**A**) Venn diagram illustrating the intersection between the putative targets of the core active components and AKI-related disease targets. (**B**) The “Disease-Pathway-Target-Component” multidimensional interaction network. Green circular nodes represent the core active components of *A*. *graminifolia*, the red triangular node represents cisplatin-induced AKI, pink square nodes represent the overlapping target genes, and yellow triangular nodes represent the signaling pathways. Node size is positively correlated with its topological degree. (**C**) Bar chart of Gene Ontology (GO) enrichment analysis categorized into Biological Process (BP), Cellular Component (CC), and Molecular Function (MF). (**D**) Bubble plot of KEGG pathway enrichment analysis. In subfigures (**C**,**D**), bubble size represents the number of enriched genes, and the color gradient (green to red) indicates statistical significance (−log10(*p*-value)). Red borders in subfigure (**D**) highlight the key signaling pathways discussed in this study.

Subsequently, the SMILES structures of these 18 active components were submitted to the SwissTargetPrediction platform. The species parameter was set to *Homo sapiens*, and a Probability score > 0.1 was applied as the threshold to acquire highly credible potential targets. Submitting these structures to SwissTargetPrediction yielded 379 potential drug targets. Intersecting these with 2640 AKI-related disease targets (retrieved from GeneCards, DisGeNET, and OMIM) resulted in 124 overlapping target genes (Figure 12A).

#### 2.5.2. “Disease-Pathway-Target-Component” Multi-Dimensional Network Topological Analysis

An interaction network was constructed using Cytoscape (Figure 12B). Compounds ranking high in degree values primarily included kaempferol, apigenin, isorhamnetin, Sinensetin and (Z)-5,8,11-trihydroxyoctadec-9-enoic acid. The polyhydroxy structural features of these flavonoids confer superior ROS scavenging abilities, helping block the oxidative stress cascade in renal tubules. The most significantly affected key targets included AKT1, PIK3R1, EGFR, PIK3CA, and PIK3CB, suggesting that BYJ’s core mechanism likely involves combating tubular cell apoptosis.

#### 2.5.3. GO and KEGG Pathway Enrichment Analysis

GO enrichment analysis (Figure 12C) showed that biological processes (BP) were concentrated in the positive/negative regulation of apoptosis, inflammatory response regulation, and oxidative stress response, highly consistent with the in vivo phenotypic improvements. KEGG pathway analysis (Figure 12D) further confirmed that the core signaling cascades were highly enriched in the PI3K-Akt signaling pathway (hsa04151), the TNF signaling pathway (hsa04668), and the MAPK signaling pathway (hsa04010).

#### 2.5.4. Computer-Aided Molecular Docking Verification

**Figure 13:**
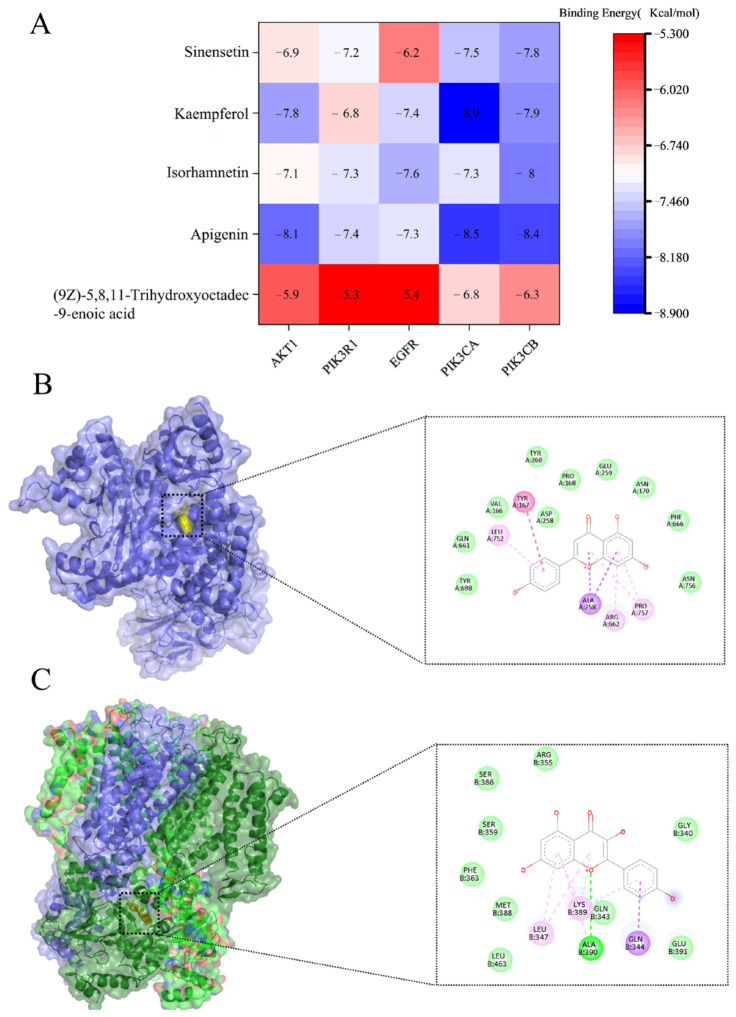
Molecular docking analysis between the core active components and key kinase targets. (**A**) The heatmap illustrating the binding energies (kcal/mol) of the kidney-recruited components against the top five hub target proteins. (**B**) The 3D binding mode and 2D interaction details of kaempferol with the active pocket of PIK3CA. (**C**) The 3D binding mode and 2D interaction details of apigenin with the active pocket of AKT1.

To validate the binding capacity between BYJ’s core active components and key AKI targets at the spatial structure level, the top 5 core components were docked against 5 key kinase targets. As shown in Figure 13, the binding energies were all below −5.3 kcal/mol, demonstrating excellent molecular recognition and spatial matching.

Flavonoid components (apigenin and kaempferol) performed exceptionally well, with binding energies generally ranging from −7.3 to −8.9 kcal/mol against AKT1, EGFR, and PI3K family proteins. Notably, the binding energy of kaempferol with PIK3CA reached −8.9 kcal/mol, and apigenin with AKT1 was −8.1 kcal/mol. These polyhydroxy flavonoids can stably anchor within the active pockets of target kinases, forming robust hydrogen bonds and hydrophobic networks with surrounding amino acid residues, providing thermodynamic structural validation for the network predictions. To further elucidate the binding mechanisms, we analyzed the detailed spatial interactions between the core components and their targets using 2D interaction diagrams. Notably, the flavonoid skeleton of kaempferol anchored deeply into the active pocket of PIK3CA primarily through robust π-interactions (such as π-alkyl and π-π stacking) with key residues including Tyr167, Leu752, Ala758, Arg662, and Pro757. Additionally, extensive van der Waals forces with surrounding residues (e.g., Tyr260, Asp258, and Gln661) further stabilized the complex, contributing to its exceptional binding affinity (−8.9 kcal/mol). Similarly, apigenin exhibited a high affinity (−8.1 kcal/mol) for AKT1. The interaction analysis revealed that apigenin formed a critical conventional hydrogen bond with the Ala390 residue. Furthermore, it engaged in strong π-interactions with Lys389, Leu347, and Gln344, supplemented by a broad network of hydrophobic contacts with residues such as Met388, Phe363, and Ser359 (Figure 13).

## 3. Discussion

Acute kidney injury (AKI) is a critical clinical condition with a high mortality rate. Its core pathological process is intertwined with oxidative stress, an inflammatory cascade storm, and extensive apoptosis of renal tubular epithelial cells. Cisplatin, as a typical chemotherapeutic agent that induces AKI, has severe nephrotoxicity that strictly limits its dosage in clinical anti-tumor applications [20]. Traditional Chinese medicine (TCM) and ethnic minority medicines, relying on their “multi-component, multi-target, multi-pathway” systemic regulatory advantages, have [21,22]. However, research on the material basis of TCM has long been plagued by two pain points: first, a disconnect exists between in vitro components and the actual in vivo functional entities, meaning that components with high abundance in vitro may not necessarily reach the target organs; second, conventional network pharmacology predictions lack authentic pharmacokinetic constraints, and directly inputting all components often leads to “false-positive” predictions. By constructing an in vivo mass spectrometry cross-analysis matrix of “Normal vs. Pathological,” this study observed and validated the “pathological targeted recruitment” characteristics of the active components of BYJ in the damaged target organs. By driving network pharmacology mechanism analysis with authentic targeted exposure components, this study provides objective evidence for establishing the pharmacological entities of TCM.

### 3.1. Specific Molecular Recruitment Characteristics in the Injured Kidneys

The pharmacodynamic results of this study indicated that the BYJ extract effectively ameliorated cisplatin-induced renal failure and microscopic histological damage. To identify the specific molecules exerting renoprotective effects locally at the lesion, this study employed high-sensitivity UPLC-Q-TOF-MS/MS to trace the in vivo distribution rules of the exogenous components.

In traditional cognition, the tissue distribution of small-molecule drugs mainly depends on organ blood flow and their own lipophilicity. However, this study observed a significant dynamic shift in distribution: under normal physiological conditions, the renal microvascular barrier exerted non-specific filtration, allowing only trace amounts of 6 BYJ prototype components to be taken up; yet, in the cisplatin-induced AKI pathological state, the number of prototype components entering the kidneys significantly increased to 16. This regional chemotaxis and accumulation phenomenon of specific drug molecules highlights a distribution characteristic of “Pathological Targeted Recruitment.” This phenomenon is likely determined synergistically by the specific pathological microenvironment of AKI and the physicochemical properties of the molecules. Cisplatin’s toxic attack causes renal microcirculatory impairment and abnormally increased microvascular permeability (i.e., the pathological leakage effect). More critically, the severe oxidative stress (ROS burst) induces massive lipid peroxidation of the tubular epithelial cell membranes, destroying the integrity of the dense lipid bilayer structure [23,24]. This study found that the specifically recruited molecules in the damaged kidneys were highly concentrated in flavonoid aglycones such as kaempferol, apigenin, and isorhamnetin. These polyhydroxy flavonoid aglycones possess strong lipophilicity and rigid planar skeletons, and the diseased microenvironment may endow them with high “pathological affinity.” They may physically embed into and stabilize the damaged and loose lipid bilayer via steric hindrance effects, thereby helping to maintain the stability of the tubular microscopic architecture. This microenvironment-driven targeted distribution provides pharmacokinetic theoretical support for understanding the localized intervention of TCM active molecules at the lesion. Additionally, while our mechanistic framework primarily focuses on the kidney-targeted components, the differential components uniquely identified in the serum (but not in the kidneys) should not be overlooked. These circulating metabolites (e.g., specific phenolic acids and highly polar glycosides) may not directly penetrate the renal barrier, but they could putatively exert systemic anti-inflammatory effects, modulate gut microbiota, or serve as circulating antioxidant reservoirs. However, as the core objective of this study was to pinpoint the direct pharmacological effectors at the lesion site, downstream network pharmacology and molecular docking were strictly limited to the authentic kidney-migrating components.

### 3.2. Significance of the “Glycoside–Aglycone” Conversion Pathway for Targeted Exposure

In addition to the direct distribution of prototype components, this study also highlighted the dynamic in vivo biotransformation trajectory of BYJ through MS fragmentation rules and metabolic correlation analysis. The raw herb of BYJ contains a large number of highly water-soluble macromolecular flavonoid O-glycosides (such as Quercetin-3-O-rutinoside). Due to their high polarity and large molecular weight, these components are generally difficult to directly penetrate the gastrointestinal mucosal barrier. The in vitro and in vivo MS correlation analysis suggests that after oral administration, these macromolecular glycosides may undergo deglycosylation reactions under the action of the gut microecology (such as β-glucosidases secreted by gut microbiota), converting into highly lipophilic free aglycones (e.g., quercetin, kaempferol) that enter the bloodstream.

Upon entering systemic circulation, these aglycone molecules not only cross the damaged renal tubular capillary barrier more easily but are also prone to generating glucuronide conjugates under the catalysis of abundant UGTs (uridine 5’-diphospho-glucuronosyltransferases) in organs like the liver and kidneys. Related studies indicate that at the target sites of inflammation and tissue damage (such as the local AKI kidney), highly expressed β-glucuronidase may promote a “deconjugation reaction,” releasing active free aglycones in situ. This delivery rule of “gut metabolism → systemic absorption → targeted recruitment → local release” suggests that the flavonoid aglycones and their metabolites, which exhibit high exposure features locally at the lesion, are likely the substantive material basis for BYJ’s efficacy. It is important to emphasize that while this dynamic in vivo biotransformation trajectory is strongly supported by our MS fragmentation rules and current literature, these convergent metabolic pathways remain proposed hypotheses. Precise enzymatic assays and microbiome perturbation models are warranted in future studies for direct mechanistic confirmation.

### 3.3. Predicted Multi-Pathway Anti-Apoptotic and Anti-Inflammatory Mechanisms Associated with Authentic Kidney-Migrating Components

To overcome the potential “false-positive” predictions in traditional network pharmacology caused by inputting total in vitro components, this study strictly defined the data input boundaries. Only the 18 targeted migrating core components (traced back to parent aglycones from metabolites) actually recruited in the damaged kidneys were used for mechanism deduction. This research strategy ensures that the subsequent network predictions and docking results possess a higher logical coherence with the body’s true pathological state. Target network and pathway enrichment (GO/KEGG) analyses indicated that the core mechanism of BYJ migrating components against AKI primarily focuses on the PI3K-Akt signaling pathway and the inflammatory cascade network dominated by TNF/MAPK [25,26]. Computer-aided molecular docking simulations further supported this analysis: the core aglycones locally recruited in the kidneys (such as kaempferol and apigenin) exhibited excellent spatial compatibility and low binding energies (−8.1 to −8.9 kcal/mol) with key kinases (AKT1, PIK3CA, EGFR) in the intervention network. Previous mechanistic studies have indicated that the overactivation of MAPK pathways is a crucial driver inducing tubular cell apoptosis [27,28]. The multi-components in BYJ may synergistically target these pathways to regulate Akt activation, thereby interfering with downstream pro-apoptotic proteins such as Bax and Bad [29].

The core molecular events of cisplatin-induced AKI include mitochondrial dysfunction and the upregulated expression of pro-apoptotic proteins like Bax. The molecular docking results suggest that kaempferol and apigenin, relying on their abundant phenolic hydroxyl groups, can form stable hydrogen bonds and hydrophobic networks with key amino acids inside the active pockets of target proteins (such as AKT1) [30,31]. Such binding may help regulate the phosphorylation activation state of Akt, further affecting the functions of downstream pro-apoptotic factors like Bad and Bax, thereby somewhat halting the apoptosis of tubular epithelial cells. Simultaneously, acting as natural antioxidants, these flavonoid aglycones are hypothesized to locally scavenge ROS at the damaged site (which aligns with the recovery of renal SOD activity observed in the animal experiments) [32], which is predicted to help block the MAPK and TNF-α inflammatory storms mediated by oxidative stress [33]. This dual mechanism of physical cell membrane protection combined with targeted regulation of kinase cascades provides a reference for deciphering the systemic pharmacological essence of BYJ’s “detoxification and renoprotection”.

### 3.4. Limitations and Future Perspectives

While this study innovatively reveals the “pathological targeted recruitment” of BYJ via high-sensitivity LC-MS/MS, several limitations warrant acknowledgment. First, the kidney-migrating components were identified using semi-quantitative MS profiling. Future targeted LC-MS/MS analysis is required for absolute quantification to verify if these recruited aglycones reach pharmacologically effective concentrations in the renal parenchyma. Second, the proposed PI3K-Akt and MAPK mechanisms rely primarily on in silico predictions. Since the in vivo samples were fully consumed for MS mapping, direct protein-level verification (e.g., Western Blotting) is currently lacking. We plan to experimentally validate these kinase cascades using in vitro HK-2 cell models and gene-silencing techniques in follow-up studies. Third, strictly following biochemical assay protocols to prevent chemical interference, kidney homogenates were prepared in cold saline without protease inhibitors, which carries a risk of partial protein degradation. Subsequent molecular validations will address this by utilizing comprehensive lysis buffers. Despite these limitations, defining the material basis based on authentic target-organ distribution provides a robust scientific foundation for the clinical translation of BYJ against AKI.

## 4. Materials and Methods

### 4.1. Reagents, Reference Standards, and Animals

Cisplatin (purity ≥ 99.0%) was purchased from Wuhan Servicebio Technology Co., Ltd. (Wuhan, China). LC-MS grade solvents (acetonitrile, methanol) and formic acid were obtained from Merck (Darmstadt, Germany) and Fisher Scientific (Waltham, MA, USA). Assay kits for serum creatinine (Scr), blood urea nitrogen (BUN), superoxide dismutase (SOD) and glutathione (GSH) were provided by Nanjing Jiancheng Bioengineering Institute (Nanjing, China). Reference standards, including resveratrol, pterostilbene, kaempferol, isorhamnetin, and vitexin (all with HPLC purity ≥ 98%), were purchased from Chengdu Must Bio-Technology Co., Ltd. (Chengdu, China).

Specific pathogen-free (SPF) male Kunming (KM) mice (4 weeks old, 22 ± 1 g) were provided by Speford (Beijing, China) Biotechnology Co., Ltd. [License No.: SCXK (Jing) 2024-0001]. The mice were housed in a standard barrier environment (22 ± 2 °C, relative humidity 55 ± 5%, 12 h light/dark cycle) with ad libitum access to food and water. All animal experiments were approved by the Institutional Animal Care and Use Committee (Approval No.: 20260106071).

### 4.2. Preparation of Arundina graminifolia Extract (BYJ)

The dried whole plant of *A. graminifolia* was purchased from Yunnan Huangya Dai Pharmaceutical Co., Ltd. (Xishuangbanna, China) and authenticated by Researcher Guang Li from the Institute of Medicinal Plant Development, Chinese Academy of Medical Sciences.

Based on our preliminary experiments [7] and the general principles of natural product extraction [34], 50% aqueous methanol was selected as the extraction solvent to provide an optimal polarity balance, ensuring the comprehensive extraction of both hydrophilic (e.g., flavonoid glycosides) and lipophilic (e.g., flavonoid aglycones) constituents. As reported in prior studies, 50% aqueous methanol shows excellent performance in extracting plant polyphenols [34]. Briefly, the whole herb was crushed and passed through a 60-mesh sieve. Precisely 500.0 g of the powder was weighed and mixed with this solvent at a solid-liquid ratio of 1:10 (g/mL). The mixture was subjected to ultrasonic extraction (300 W, 40 kHz) for 30 min at room temperature. The extract was filtered, and the solvent was recovered under reduced pressure at 50 °C. The concentrated extract was then lyophilized at −80 °C to yield the *A. graminifolia* extract (BYJ) dry powder, which was sealed and stored in the dark for future use.

### 4.3. Establishment of the AKI Animal Model and Intervention Protocol

The cisplatin-induced mouse model is widely recognized for accurately recapitulating the core clinical and pathological features of human AKI, including extensive renal tubular necrosis, oxidative stress bursts, and inflammatory cascades [35]. In this study, Kunming (KM) mice were selected as the experimental subjects. As a widely used outbred strain, KM mice possess a high degree of genetic heterogeneity. Compared to highly inbred strains (such as C57BL/6), this genetic diversity better mimics the heterogeneous genetic background of human populations, making them highly suitable for evaluating the general pharmacological efficacy of complex traditional medicines [36]. Furthermore, previous studies have shown that strain background significantly influences susceptibility to cisplatin-induced nephrotoxicity, and KM mice have been successfully employed to establish stable and reproducible AKI models [19].

After one week of adaptive feeding, the mice were randomly divided into four groups (*n* = 6 per group): the blank control group (Control), the BYJ normal administration group (BYJ-Normal), the AKI model group (Model), and the BYJ treatment group (BYJ-Model).

The lyophilized BYJ powder was suspended in 0.5% sodium carboxymethyl cellulose (CMC-Na). Based on our previously published study demonstrating its optimal in vivo renoprotective efficacy and a clear dose-response relationship [37], BYJ was intragastrically administered to the BYJ-Normal and BYJ-Model groups at a dose of 8 g/kg (expressed as crude herb equivalent, volume: 10 mL/kg) daily for 10 consecutive days. This 10-day administration window was strategically designed to encompass a 7-day preventive pre-treatment phase and a 3-day therapeutic phase post-induction. This duration ensures sufficient systemic exposure prior to the challenge and covers the 72-h period during which cisplatin-induced nephrotoxicity typically reaches its pathological and biochemical peak (Day 10). The Control and Model groups received an equal volume of 0.5% CMC-Na via intragastric administration. Two hours after administration on the 7th day, the Model and BYJ-Model groups received a single intraperitoneal injection of cisplatin (13 mg/kg, freshly prepared in physiological saline and protected from light) to induce AKI. The specific cisplatin dosage of 13 mg/kg was optimized through our preliminary experiments; compared to higher doses that often cause severe mortality, this optimized dose reliably induces acute tubular necrosis and oxidative stress while maintaining a stable survival rate throughout the observation period. The other two groups were injected with an equal volume of physiological saline. A comprehensive schematic illustration detailing the animal grouping, dosing regimen, and sampling timeline is provided in the Supplementary Materials (Supplementary Figure S1).

### 4.4. Sample Collection and Biochemical/Histopathological Evaluation

One hour after the final drug administration on the 10th day, blood was collected via eyeball extirpation. The blood was allowed to stand at 4 °C for 2 h and then centrifuged (15,800× *g*, 10 min) to separate the serum, which was stored at −80 °C. The mice were euthanized by cervical dislocation. Bilateral kidneys were excised and weighed to calculate the kidney index (kidney weight/body weight × 100%).

Biochemical analysis: Serum Scr and BUN concentrations were measured using a Hitachi 7180 automatic biochemical analyzer (Hitachi, Tokyo, Japan). The right kidney was homogenized with cold physiological saline to prepare a 10% (*w*/*v*) tissue homogenate. This specific homogenization medium, devoid of additional lysis buffers or protease inhibitors, was strictly chosen according to the assay kits’ manufacturer instructions to prevent any chemical interference with the subsequent colorimetric reactions. After centrifugation, the supernatant was collected to determine SOD activity and GSH content according to the manufacturers’ instructions, with normalization by total protein concentration using the BCA method.

Histopathology: The left kidney was fixed in 4% paraformaldehyde for 24 h. Following routine dehydration and paraffin embedding, the tissues were sectioned (4 μm) and stained with hematoxylin and eosin (H&E). Renal tubular damage was observed under a light microscope (Olympus, Tokyo, Japan) 200×).

### 4.5. UPLC-Q-TOF-MS/MS Sample Preparation and Analytical Conditions

Sample preparation: A 200 μL aliquot of serum was mixed with 600 μL of methanol-acetonitrile (1:1, *v*/*v*) to precipitate proteins, vortexed, and centrifuged at 4 °C (18,600× *g*, 10 min). The supernatant was evaporated to dryness under nitrogen at 30 °C, reconstituted in 100 μL of 10% acetonitrile, and filtered prior to analysis. For kidney tissues, 20 mg of the sample was thoroughly homogenized in 100 μL of 0.1% aqueous formic acid. Subsequently, to precipitate proteins and extract the metabolites, 300 μL of cold methanol-acetonitrile (1:1, *v*/*v*) was added to the homogenate. The mixture was vortexed for 3 min and then centrifuged at 4 °C (18,600× *g*, 10 min). The supernatant was carefully collected, dried under nitrogen at 30 °C, reconstituted in 200 μL of 0.1% aqueous formic acid, and filtered through a 0.22 μm membrane prior to analysis.

Chromatographic conditions: Separation was performed on an Acquity UPLC BEH C18 column (100 × 2.1 mm, 1.7 μm). The mobile phases consisted of 0.1% aqueous formic acid (A) and 100% acetonitrile (B). The gradient elution program was as follows: 0–2 min, 5% B; 2–4 min, 5–55% B; 4–5 min, 55–70% B; 5–13 min, 70–82.5% B; 13–14 min, 82.5–95% B; 14–19 min, 95% B; 19–19.1 min, 95–5% B; 19.1–22 min, 5% B. The flow rate was 0.3 mL/min, the injection volume was 2 μL, and the column temperature was 40 °C.

Mass spectrometry conditions: An AB SCIEX X500B Q-TOF-MS/MS instrument(AB Sciex, Framingham, MA, USA) equipped with an electrospray ionization (ESI) source was used. Scanning was performed in both positive and negative ion modes, utilizing the Information Dependent Acquisition (IDA) mode and Dynamic Background Subtraction (DBS) algorithm. The parameters were set as follows: ISVF ±5500 V; TEM 550 °C; GS1 and GS2 both at 60 psi; CUR 35 psi; DP 100 V; CE 40 V with CES 20 V. The full scan range was *m*/*z* 100–1200. During the analysis, an automated Calibration Delivery System (CDS) was employed for continuous mass calibration to ensure high mass accuracy, maintaining the mass error strictly within 5 ppm. It should be noted that no internal standard was used in this untargeted qualitative analysis, as the primary objective was to identify the presence of migrating components rather than perform absolute quantification. To ensure system stability and data reproducibility, the instrument performance was verified daily using the manufacturer’s standard calibration solution, and the consistency of the chromatographic peak shapes and retention times was closely monitored throughout the entire sequence.

### 4.6. MS-DIAL-Based Metabolite Identification Strategy

Raw mass spectrometry data were imported into MS-DIAL (v4.9) for peak extraction and alignment. The data filtering strategy was as follows:

(1) Background subtraction: Feature peaks with a signal response (Peak Area) > 5 times that of the blank group and a signal-to-noise ratio (S/N) ≥ 10 in the administration group were extracted.
(2) Specificity screening: By comparing the chromatograms of the Model and BYJ-Model groups, the core component clusters pathologically recruited to the kidneys in the AKI state were identified.
(3) Structural matching: With a mass error limit of < 5 ppm, the MS/MS fragmentation patterns were matched against reference standards and online databases (HMDB, MassBank, METLIN), requiring a matching score ≥ 80%. Based on classical Phase I/II drug metabolism rules, the in vivo metabolic network was delineated.

### 4.7. Network Pharmacology and Molecular Docking

Network construction and enrichment analysis: The SMILES structures of the identified core kidney-migrating components were submitted to SwissTargetPrediction for target prediction. A threshold of Probability > 0.1 was applied to exclude low-confidence targets while retaining structurally relevant interactions [38,39]. Concurrently, AKI-associated targets were retrieved from the GeneCards, DisGeNET, and OMIM databases. For the GeneCards database, a Relevance score > 1.0 was strictly set to eliminate loosely associated background targets and ensure strong clinical and experimental relevance [40]. These empirical thresholds were selected based on established network pharmacology strategies to optimize the balance between analytical sensitivity and specificity. After obtaining the intersecting genes using Venny 2.1, an interaction network was built using Cytoscape 3.10.1. The overlapping genes were imported into the DAVID database (v6.8) for Gene Ontology (GO) and Kyoto Encyclopedia of Genes and Genomes (KEGG) enrichment analyses (*p* < 0.05).

Molecular docking: The 2D structures of the ligands and the 3D crystal structures of the receptors (AKT1, PIK3CA, EGFR) were obtained from the PubChem and RCSB PDB databases, respectively. The protein preparation steps were rigorously performed using PyMOL (v2.4) to remove water molecules and original co-crystallized ligands. Subsequently, the dehydrated proteins were imported into AutoDockTools (v1.5.6) to add polar hydrogens and compute Gasteiger charges. For the docking simulation, a grid box of 20 × 20 × 20 Å was established, with the coordinates centered exactly on the binding site of the original co-crystallized ligands to accurately define the active pockets. The docking calculations were executed using AutoDock Vina (v1.1.2) with the exhaustiveness parameter set to 8. The docking poses with the lowest binding energies (kcal/mol) were recorded and selected for 2D and 3D visualization.

### 4.8. Statistical Analysis

All quantitative data were derived from at least three independent experiments and are expressed as mean ± standard deviation (SD). Statistical analyses were performed using GraphPad Prism 8.0 and SPSS 27.0 software. For data conforming to a normal distribution (evaluated by the Shapiro-Wilk test) and homoscedasticity (assessed by Levene’s test), a one-way analysis of variance (ANOVA) followed by Tukey’s post-hoc test was applied. Non-normally distributed data were analyzed using the Kruskal-Wallis test followed by Dunn’s post-hoc test for multiple comparisons. A *p*-value < 0.05 was considered statistically significant.

## 5. Conclusions

By integrating in vivo target organ mass spectrometry tracking with network pharmacology prediction, this study explored the action mechanisms and pharmacodynamic material basis of the Dai medicine *A. graminifolia* (BYJ) in intervening against cisplatin-induced acute kidney injury (AKI). Animal experiments verified that the BYJ extract effectively restored renal antioxidant capacity and alleviated renal tubular damage. Through in vitro and in vivo mass spectrometry cross-analysis, this study discovered a significant distribution shift: under the influence of the damaged AKI microenvironment, highly lipophilic flavonoid aglycones, predominantly kaempferol and apigenin, achieved specific targeted recruitment locally in the kidneys. Network pharmacology and molecular docking analyses based on authentic kidney-migrating components strongly suggested that this core cluster possesses high binding affinities to key targets such as AKT1 and PIK3CA, potentially exerting anti-apoptotic and anti-inflammatory effects primarily by regulating the PI3K-Akt and TNF signaling pathways. This study elucidated the material basis of BYJ from the perspective of actual in vivo distributed components, clarified the rationale for using kaempferol and apigenin as key quality control markers, and provided objective experimental evidence for related research on the action mechanisms of TCM.

## Figures and Tables

**Table 1:** Comprehensive summary of the identified prototype components and their representative metabolites in vivo, including their metabolic pathways and distribution under normal and AKI states.

No.	Prototype Name	Metabolite Name	Type	Metabolic Pathway	Class	Serum (BYJ-Normal)	Kidney (BYJ-Normal)	Serum (BYJ-Model)	Kidney (BYJ-Model)
1	Kaempferol	Kaempferol	Prototype	Intact intestinal absorption	Flavonoid	-	-	-	+
2	Kaempferol-3-O-glucoside	Kaempferol-3-O-glucoside	Prototype	Intact intestinal absorption	Flavonoid	+	-	-	+
3	Kaempferol-3-O-α-L-rhamnoside	Kaempferol-3-O-α-L-rhamnoside	Prototype	Intact intestinal absorption	Flavonoid	+	-	-	-
4	Kaempferol	Dihydrokaempferol	Metabolite	Phase I reduction (intestinal flora)	Flavonoid	-	+	-	-
5	Kaempferol	Camelliaside A	Metabolite	Phase II glycosylation	Flavonoid	+	-	+	-
6	Kaempferol	Kaempferol 3-glucuronide	Metabolite	Phase II glucuronidation (UGTs)	Flavonoid	+	-	+	-
7	Kaempferol	Kaempferol-3-O-glucuronide	Metabolite	Phase II glucuronidation (UGTs)	Flavonoid	-	-	-	+
8	Kaempferol 3-rutinoside 4’-glucoside	Mauritianin	Metabolite	Phase I deglycosylation (loss of sugar moiety)	Flavonoid	+	-	+	-
9	Kaempferol 3-rutinoside 4’-glucoside	Kaempferol-7-O-glucoside	Metabolite	Phase I deglycosylation (loss of sugar moiety)	Flavonoid	-	+	-	+
10	Apigenin	Apigenin	Prototype	Theoretical aglycone mapping	Flavonoid	-	-	-	-
11	Apigenin	Acacetin	Metabolite	Phase II O-methylation (COMT)	Flavonoid	+	-	+	-
12	Apigenin	Baicalin	Metabolite	Phase I hydroxylation + Phase II glucuronidation	Flavonoid	+	-	+	-
13	Apigenin	Apigenin 7-O-glucuronide	Metabolite	Phase II glucuronidation (UGTs)	Flavonoid	-	-	-	+
14	Apigenin	Tectochrysin	Metabolite	Phase I dehydroxylation + Phase II methylation	Flavonoid	-	-	+	+
15	Apigenin	5-hydroxy-2-(4-hydroxyphenyl)-6,7-dimethoxychromen-4-one	Metabolite	Phase I hydroxylation + Phase II O-methylation	Flavonoid	-	+	-	-
16	Quercetin	Quercetin	Prototype	Intact intestinal absorption	Flavonoid	+	-	+	-
17	Quercetin	Quercetin 3-O-glucuronide	Metabolite	Phase II glucuronidation (UGTs)	Flavonoid	-	+	-	-
18	Quercetin	3-O-methylquercetin	Metabolite	Phase II O-methylation (COMT)	Flavonoid	-	-	+	-
19	Isorhamnetin	Isorhamnetin	Prototype	Intact intestinal absorption	Flavonoid	+	-	+	+
20	Isorhamnetin-3-O-rutinoside	Isorhamnetin-3-O-rutinoside	Prototype	Intact intestinal absorption	Flavonoid	-	-	-	+
21	Isorhamnetin 3-galactoside	Isorhamnetin 3-galactoside	Prototype	Intact intestinal absorption	Flavonoid	+	-	+	-
22	Isorhamnetin-3,7-di-O-glucoside	Isorhamnetin 3-sophoroside-7-rhamnoside	Metabolite	Phase II transglycosylation	Flavonoid	+	-	+	-
23	Isorhamnetin	(2S)-5,7-dihydroxy-2-(3-hydroxy-4-methoxyphenyl)-2,3-dihydro-4H-chromen-4-one	Metabolite	Phase I reduction (intestinal flora)	Flavonoid	+	-	+	-
24	Quercetin-3-O-rutinoside	Quercetin-3-O-rutinoside	Prototype	Intact intestinal absorption	Flavonoid	-	-	-	+
25	Sinensetin	Sinensetin	Prototype	Intact intestinal absorption	Flavonoid	+	-	+	+
26	Sinensetin	Nobiletin	Metabolite	Phase I hydroxylation + Phase II O-methylation	Flavonoid	+	-	+	+
27	Sinensetin	Isosinensetin	Metabolite	Phase I structural isomerization	Flavonoid	+	-	+	-
28	Sinensetin	Artemitin	Metabolite	Multi-step phase I & II biotransformation	Flavonoid	-	-	-	+
29	Resveratrol	Resveratrol	Prototype	Theoretical aglycone mapping	Stilbene	-	-	-	-
30	Resveratrol	Triacetylresveratrol	Metabolite	Phase II acetylation	Stilbene	-	-	-	+
31	Luteolin 6-C-glucoside 8-C-arabinoside	Luteolin	Metabolite	Phase I deglycosylation (loss of sugar moiety)	Flavonoid	-	+	-	-
32	Luteolin 6-C-glucoside 8-C-arabinoside	Luteolin 7-glucuronide	Metabolite	Phase I deglycosylation + Phase II glucuronidation	Flavonoid	-	+	-	-
33	Biochanin A	Biochanin A 7-O-glucoside	Metabolite	Phase II glycosylation	Flavonoid	-	-	+	-
34	Delphinidin 3-galactoside	Malvidin	Metabolite	Phase I deglycosylation + Phase II O-methylation	Flavonoid	-	-	+	-
35	5,6,7-trimethoxy-2-(4-methoxyphenyl)chromen-4-one	5,6,7-trimethoxy-2-(4-methoxyphenyl)chromen-4-one	Prototype	Intact intestinal absorption	Flavonoid	+	-	+	-
36	5,6,7-trimethoxy-2-(4-methoxyphenyl)chromen-4-one	5-hydroxy-2-(4-hydroxyphenyl)-6,7-dimethoxychromen-4-one	Metabolite	Phase I O-demethylation (CYP450)	Flavonoid	-	-	-	+
37	Vitexin	Apigenin 6,8-digalactoside	Metabolite	Phase I dephosphorylation	Flavonoid	-	+	-	-
38	(Z)-5,8,11-trihydroxyoctadec-9-enoic acid	(Z)-5,8,11-trihydroxyoctadec-9-enoic acid	Prototype	Intact intestinal absorption	Organic ac	-	+	+	+
39	4-Coumarate	4-Coumarate	Prototype	Intact intestinal absorption	Organic ac	-	-	-	+
40	Trans-Cinnamic acid	Trans-Cinnamic acid	Prototype	Intact intestinal absorption	Organic ac	-	+	+	+
41	4-Hydroxybenzoic acid	4-Hydroxybenzoic acid	Prototype	Intact intestinal absorption	Organic ac	+	+	-	-
42	Catechol	Catechol	Prototype	Intact intestinal absorption	Organic ac	+	+	+	-
43	L-Tyrosine	L-Tyrosine	Prototype	Intact intestinal absorption	Amino acid	+	-	+	+
44	Tryptophan	Tryptophan	Prototype	Intact intestinal absorption	Amino acid	+	+	+	+
45	N-Fructosyl pyroglutamate	L-Pyroglutamic acid	Metabolite	Phase I deglycosylation (loss of sugar moiety)	Organic ac	-	+	-	-
46	Tigogenin	Tigogenin	Prototype	Intact intestinal absorption	Terpenoid	+	-	+	+
47	Corosolic acid	Corosolic acid	Prototype	Intact intestinal absorption	Terpenoid	-	-	+	+
48	Abietic acid	Abietic acid	Prototype	Intact intestinal absorption	Terpenoid	-	-	+	-
49	Inosine	Inosine	Prototype	Intact intestinal absorption	Nucleoside	+	+	+	+
50	Benzaldehyde	Benzaldehyde	Prototype	Intact intestinal absorption	Others	+	-	-	+
51	4-Hydroxyquinoline	4-Hydroxyquinoline	Prototype	Intact intestinal absorption	Others	+	-	+	+
52	Aurantiamide	Aurantiamide	Prototype	Intact intestinal absorption	Others	-	-	+	-
53	Phytosphingosine	Phytosphingosine	Prototype	Intact intestinal absorption	Lipid	+	-	-	-
54	1-(9Z,12Z-Octadecadienoyl-2-hydroxy-sn-glycero-3-phosphocholine	1-(9Z,12Z-Octadecadienoyl-2-hydroxy-sn-glycero-3-phosphocholine	Prototype	Intact intestinal absorption	Lipid	+	-	+	-

Note: “+” indicates detected; “-” indicates not detected.

## Data Availability

The original mass spectrometry data presented in the study are included in the article/Supplementary Materials; further inquiries can be directed to the corresponding author.

## Supplementary Materials

The following supporting information can be downloaded at: https://www.mdpi.com/article/10.3390/molecules31111951/s1, Supplementary Table S1: Detailed identification of the 93 chemical components characterized in the Arundina graminifolia extract (BYJ) in vitro using UPLC-Q-TOF-MS/MS; Supplementary Table S2: Detailed information and chemical structures of the 18 therapeutic core components used as the input foundation for network pharmacology analysis; Supplementary Figure S1: Schematic illustration of the experimental design and in vivo timeline; Supplementary Figure S2: Heatmap visualization of the relative abundances of the 18 therapeutic core components in the kidneys.
