# Supplementary material for: Arundina graminifolia Ameliorates Cisplatin-Induced Acute Kidney Injury via Pathological Targeted Recruitment of Flavonoid Aglycones: A Study Integrating Serum/Kidney Pharmacochemistry and Network Pharmacology
Source: Molecules. 2026 Jun 4;31(11):1951. doi: 10.3390/molecules31111951 (PMC13257526; doi:10.3390/molecules31111951)
Supplement: Supplementary file 1 [file molecules-31-01951-s001.zip › Supplementary Figure.pdf]

Supplementary Figure

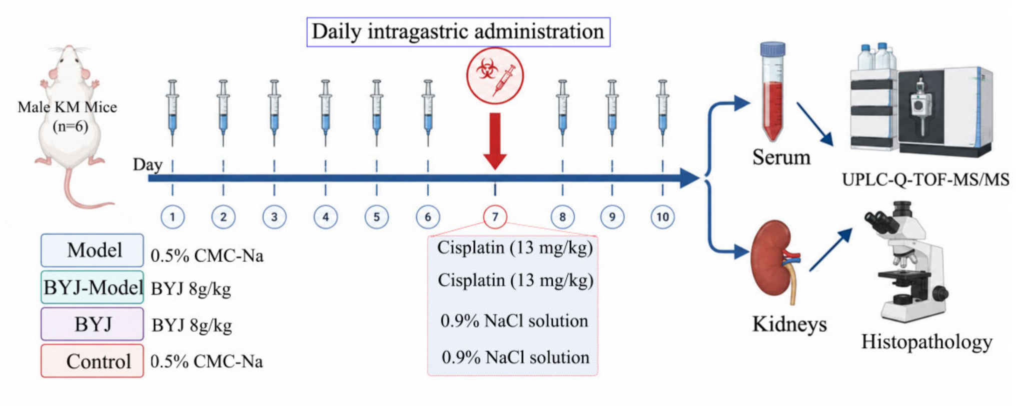

Supplementary Figure S1. Schematic illustration of the experimental design and in vivo timeline. Male KM mice (n = 6 per group) were randomly divided into four groups: Control, Model, BYJ, and BYJ-Model. The BYJ and BYJ-Model groups received daily intragastric administration of Arundina graminifolia extract (BYJ, 8 g/kg), while the Control and Model groups received an equal volume of the vehicle (0.5% CMC-Na) for 10 consecutive days. On Day 7, acute kidney injury (AKI) was induced by a single intraperitoneal injection of cisplatin (13 mg/kg) exclusively in the Model and BYJ-Model groups; the Control and BYJ groups received an equal volume of 0.9% NaCl solution. On Day 10 (1 hour post the final administration), serum and kidney samples were collected for subsequent UPLC-Q-TOF-MS/MS metabolomic profiling and histopathological evaluations.

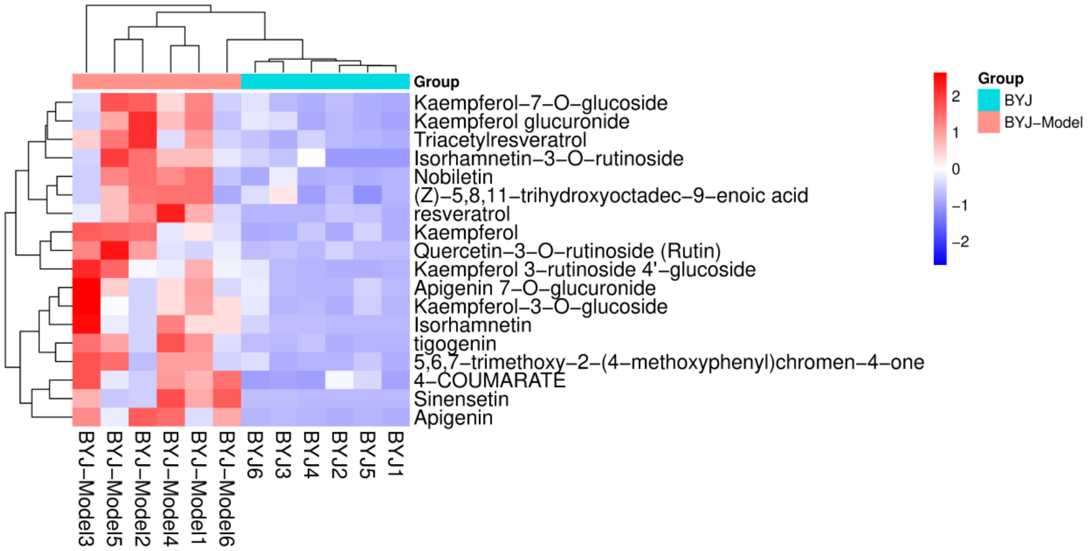

Supplementary Figure S2. Heatmap visualization of the relative abundances of the 18 therapeutic core components in the kidneys. The heatmap displays the Z-score normalized MS peak areas of the components identified in the healthy kidneys (BYJ, cyan bar) and the cisplatin-damaged kidneys (BYJ-Model, pink bar). The color scale ranges from blue (low relative abundance) to red (high relative abundance). The hierarchical clustering tree on the left indicates structural and abundance similarities among the metabolites. The distinct transition from blue to red unequivocally demonstrates the massive 'pathological targeted

recruitment' of these active components exclusively in the injured renal microenvironment.
